# The Protective Role of Resilience in the Development of Social Media Addiction in Tertiary Students and Psychometric Properties of the Slovenian Bergen Social Media Addiction Scale (BSMAS)

**DOI:** 10.3390/ijerph192013178

**Published:** 2022-10-13

**Authors:** Mark Žmavc, Andrej Šorgo, Branko Gabrovec, Nuša Crnkovič, Katarina Cesar, Špela Selak

**Affiliations:** 1 National Institute of Public Health, 1000 Ljubljana, Slovenia; 2 Faculty of Natural Sciences and Mathematics, University of Maribor, 2000 Maribor, Slovenia

**Keywords:** social media addiction, behavioral addictions, resilience, validation, Bergen Social Media Addiction Scale (BSMAS), COVID-19

## Abstract

With the onset of the COVID-19 pandemic, social media became one of the most utilized sources of information relating to the disease. With the increased reliance on social media, the risk of excessive use and the development of social media addiction emerges. The aim of the present study was to explore the psychometric properties of the Slovenian version of the Bergen Social Media Addiction Scale, and to explore how psychological resilience affects social media addiction symptoms directly and indirectly through symptoms of depression, anxiety and mental distress. A large online cross-sectional study was conducted in March 2021 among Slovenian tertiary students (*N* = 4868). The results showed the high reliability, unidimensionality and criterion validity of the Slovenian Bergen Social Media Addiction Scale. The proposed structural model fit the data well and showed a significant direct positive effect of depression and stress on social media addiction. Moreover, the majority of the negative effects of psychological resilience on social media addiction (87.2%) were indirect, through depression and stress symptoms, whereas resilience had a significantly smaller impact on social media addiction by reducing anxiety symptoms. The overall prevalence of social media addiction symptoms was 4.6%, with females exhibiting higher proportions than men. Additionally, female social media users reported a complete absence of social media addiction symptoms less often compared to males. Future research should further explore the mechanisms behind social media addiction, in order to gain a better understanding of the apparently different risk levels for both genders.

## 1. Introduction

In 2022, about 4.65 billion people worldwide used social networking platforms, commonly referred to as social media, representing around 58.7% of the world’s population [1]. Many use social media as their main source of information and form opinions and decisions based on it [2]. Social media have played an important role in the COVID-19 pandemic as the most utilized source of information on the disease [3], allowing quick dissemination of the latest knowledge and policies for reducing the spread of the virus. On the other hand, social media have also been one of the main sources of disinformation and so called “fake news,” which creates confusion and uncertainty among the population [4]. Secondly, due to limited opportunities for offline social contact during this time, social connections with others were largely maintained through social media [5]. Unsurprisingly, social media platforms reported a significant increase of both the number of users and duration of daily use during the pandemic [6]. Among the most prominent concerns regarding our reliance on social media has been the users’ tendency towards excessive use and the risk for developing social media addiction [7].

The addictive potential of social media is well known. The largest international prevalence study of social media addiction to date, conducted by Cheng et al. [8], reported global prevalence rates of 5%, 13% and even 25%, depending on the criteria used. Although the 25% figure does not refer to the number of users with clinically significant symptoms of social media addiction, it is an alarming indication of how easy it is for the average user to develop addiction-like symptoms.

Bhargava and Velasquez [9] identified and described examples of three common design elements of social media, which contribute to their addictive potential: the use of intermittent variable rewards (rewards that vary in terms of frequency or magnitude), rewards centred around our desire for social validation and reciprocity, and the erosion of natural stopping cues. The development of social media addiction then depends on many factors, as shown in a recent review of theoretical models applied in the studies of social media addiction by Sun and Zhang [7]. These factors are personality characteristics (e.g., attachment style, identity type), psychological needs and motivations (e.g., sense of belonging, flow), neurobiological characteristics (e.g., cognitive-behavioural control), maladaptive cognitions (e.g., perceived irreplaceability), psychopathologies (e.g., social anxiety), skills (e.g., deficient self-regulation) and various life circumstances (e.g., lack of social support). Due to the complexity of the phenomenon and difficulties in regulating social networking platforms, social media addiction presents an increasingly challenging public health issue [10]. Successfully addressing this issue, from identifying problematic users to offering ways out, requires validated and sensitive diagnostic tools.

Among the instruments measuring problematic social media use, the Bergen Social Media Addiction Scale (BSMAS) [11] has been one of the most commonly empirically evaluated and validated instrument in different languages and cultures [12]. BSMAS is an adaptation of the earlier Bergen Facebook Addiction Scale (BFAS) [13] with the word “Facebook” being replaced with “social media”, so that the questions refer to any social media platform, not just Facebook. The scale follows the rationale of the component model of addiction (salience, mood modification, tolerance, withdrawal, conflict, and relapse) proposed by Griffiths [14], with each component represented by one item of the BFAS. Andreassen et al. [13] report a unidimensional factor structure of BFAS and adequate factor loadings of all six items (>0.50). Additionally, internal consistency (α = 0.83) and test–retest reliability (3 weeks apart; *r* = 0.82) indicate adequate reliability. Authors found strong correlations between the BFAS and three existing measures of social media addiction. Moreover, significant correlations with personality traits were reported: high neuroticism, extraversion and low conscientiousness were associated with higher BFAS scores. Delayed bedtimes and rising times were also positively correlated to the BFAS score. Measurement invariance between genders was established on a metric level (constrained factor loadings across groups). Importantly, these results were obtained on a convenience sample of 423 Norwegian college students, indicating the need for more validation efforts across demographic groups and cultures.

Despite the minor differences between the BFAS and the BSMAS, validation of one of the two scales does not imply the validity of the other due to the possibility of some patterns of use being unique to Facebook rather than social media platforms in general. The psychometric properties of BSMAS were evaluated and published in various cultural contexts as shown in Table 1. All eight listed references report a unidimensional structure of the scale with acceptable values of model-fit indices (CFI: 0.967–0.996, RMSEA: 0.031–0.081). Internal consistency estimates (Cronbach’s α) in these studies ranged between 0.75 and 0.90, indicating adequate reliability. Measurement invariance between genders [15,16,17], age groups [17] and countries [18] was tested and (partially) confirmed in four studies. Significant positive correlations were found between the BSMAS score and frequency of social media use [12,15,18,19,20], gaming disorder [15,17,18] and symptoms of depression [12,15,19,20], anxiety [12,15,16,19] and stress [12,15,19]. The predominant focus of most validation studies was on the population of young adults and adolescents. Five out of eight studies used convenience sampling methods.

Additionally, two studies [20,21] have suggested a cut-off BSMAS score for the diagnosis of social media addiction based on empirical analysis. Bányai et al. [20] conducted a latent profile analysis on the BSMAS data and concluded that a cut-off score of 19 (out of 30) has the highest classifying accuracy for individuals “at risk” of social media addiction. However, Luo et al. [21] have conducted structured diagnostic interviews in a clinical sample to determine the presence of social media addiction. The results showed that a cut-off score of 24 has the highest overall diagnostic accuracy (98.8%), whereas a cut-off score of 19 had a relatively low proportion of correctly diagnosed positive cases (43.7%).

The present study took place during the COVID-19 pandemic and the associated governmental measures implemented in an attempt to prevent and control the spread of the SARS-CoV-2 virus (e.g., lockdown, school closures, quarantine). Several studies have shown increases in psychological distress and symptoms of mental disorders during outbreaks of infectious diseases, including the COVID-19 pandemic [22,23]. Psychological responses such as anxiety, depression and self-reported stress have been particularly common [24].

Researchers have further suggested that resilience represents a key construct predicting mental wellbeing during these times. Namely, high resilience was found to be associated with the absence of depressive and anxiety symptoms [25], and the absence of mental distress [26]. The study of resilience likely originated from the disciplines of psychology and psychiatry in the 1940s in an effort to understand the process of psychopathology development in “at risk” children [27]. One definition describes resilience as a group of personal qualities that enable one to thrive in the face of adversity [28]. A more contemporary view, however, is that the resilience of a person or a family extends beyond the individual or family-system level [29]. Many factors and systems contribute as an interactive dynamic process that increases resilience relative to adversity. Resilience may be context and time specific and may not be stable across life domains [30].

Considering that symptoms of depression, anxiety and stress were found to predict social media addiction symptoms, as reported above, it is sensible to propose that resilience may have some effect on whether a social media user develops symptoms of social media addiction or not. We expect that more resilient social media users will present with fewer symptoms of poor mental health (on average) and will therefore be less prone to the excessive and addictive use of social media.

The purpose of the present study was twofold. Firstly, our aim was to assess the psychometric properties of the Slovenian version of BSMAS in order to obtain a validated measure for assessing social media addiction in the population of Slovenian internet users. Secondly, we investigated whether resilience functions as an important predictor of social media addiction symptoms. We hypothesised that a significant part of its effect on social media addiction would be through reducing the likelihood of symptoms of depression, anxiety and stress.

## 2. Materials and Methods

### 2.1. Participants

The sample for the BSMAS validation study consisted of 4868 participants (tertiary students), who provided complete responses to all items of the five questionnaires listed in the Measures section. They represented 81.1% of the original sample (*N* = 5999). A total of 72.4% declared themselves as females (*n* = 3524), 26.8% as males (*n* = 1306) and 0.8% as other (*n* = 37). Participants’ ages ranged from 18 to 57 years, and the average age was 22.9 years (SD = 3.19 years).

In order to address the issue of potential bias due to missing data, Little’s MCAR (missing completely at random) test [31] was used to assess if missing data are positioned completely at random in the data set of interest. After inspecting the Little’s MCAR test results (χ^2^ = 29.458, df = 44, *p* = 0.955) we concluded it is safe to delete the data sets of respondents with missing data (listwise), particularly after taking the large sample size into account [32].

### 2.2. Procedure

The collection of data was a part of a larger cross-sectional study to assess mental health and its factors among the tertiary students in Slovenia during the COVID-19 pandemic. Data were obtained through a survey on the web-based survey platform 1 ka [33]. An invitation letter to participate in the study was sent to all Slovenian universities, private faculties and student organizations with a request to forward the survey to all of their students, in an effort to reach the entire tertiary student population of Slovenia. A call to participate was posted on multiple institutional webpages and their social media platforms in addition to e-mail invitations sent to students by universities and faculties. Data collection took place between 9 February and 8 March 2021. A total of 5999 full-time students responded, which represents about 10% of the entire Slovenian tertiary student population. The full set of descriptive data analyses is available online [34].

#### 2.2.1. Adaptation of the Slovenian BSMAS

The BSMAS was translated from English to Slovenian by two experts in the field of mental health and digital addiction, both Slovenian native speakers, and then additionally checked by the lead methodologists of the research project (experts with a background in psychology psychometrics and public health). The process of translation followed the three-step Eurostat translation protocol [35]. No adaptations due to cultural differences were deemed necessary.

#### 2.2.2. Statistical Analyses

The statistical methods used can be separated into two parts. The first part was an assessment of the BSMAS and consisted of the following; (i) frequencies and descriptive statistics of key variables, (ii) test of distribution normality, Keiser–Mayer–Olkin test for sampling adequacy and Bartlett’s test of sphericity, (iii) principal component analysis (PCA) to inspect potential deviation from unidimensionality of the BSMAS, (iv) confirmatory factor analysis (CFA) using robust diagonally weighted least squares (DWLS) estimation procedure [36] due to ordinal data and deviations from normality of distributions, (v) internal consistency and construct reliability, assessed by Cronbach’s alpha, (vi) Spearman correlation coefficient ρ for evaluating criterion validity, (vii) Mann–Whitney test to compare results across genders with respective effect sizes (Cohen’s *d)*, calculated from the values of *U*, and (viii) Cohen’s *h* to establish the magnitude of an effect between two proportions was calculated by application of the Excel formula: Cohen’s *h* = 2 × (ASIN(SQRT(p1)) − ASIN(SQRT(p2))).

The second part was dedicated to the testing of the structural equation model (SEM), constructed to assess the potential protective role of resilience. All latent constructs included in the model were subjected to CFA and reliability assessment. The DWLS estimation procedure was applied for both—measurement and structural models. The only model modification procedure was constraining residual error terms within a construct (latent variable) following the suggestions of the JAMOVI 2.3.12 software [37]. The magnitude of indirect effects was calculated by the multiplication of values of direct path coefficients as suggested by Kline [38].

All the statistical procedures were performed using the JAMOVI software, version 2.3.12 [37] and by Psychometrica, an online tool for the calculation of effect sizes [39].

### 2.3. Measures

The structured web-based survey included measures of social media addiction, resilience, and symptoms of stress, anxiety, and depression. The original versions of these measures have been well established and validated, with the latter four being validated in the Slovenian language by Gabrovec et al. [34].

#### 2.3.1. Social Media Addiction

The Bergen Social Media Addiction Scale (BSMAS) [11] comprises six items regarding a person’s relationship with and use of social media, with the latter being defined as “Facebook, Twitter, Instagram, and the like”. Answers are given on a 5-point Likert scale ranging from very rarely (1) to very often (5), which results in a total score between 6 and 30. Higher scores are associated with a higher risk of social media addiction. Questions refer to experiences during the past year (e.g., “How often during the last year have you used social media to forget about personal problems”). The scale has been translated into several languages (see Section 1) with consistent reports of a unidimensional factor structure. The reliability of the Slovenian version of BSMAS was 0.87 in the present study.

#### 2.3.2. Resilience

Resilience, i.e., an individual’s ability to cope with stress and adversity, was assessed with the Connor–Davidson Resilience Scale (CD-RISC) [28]. Specifically, the 10-Item Connor–Davidson Resilience Scale, adapted by Cambell-Sills and Stein [40], which demonstrated good internal consistency and construct validity, was used in the study due to its brevity. Items of the scale correspond to the respondent’s flexibility (items 1 and 5), sense of self-efficacy (items 2, 4 and 9), ability to regulate emotion (item 10), optimism (items 3, 6 and 8) and cognitive focus under stress (item 7). Items are assessed on a 5-point Likert Scale ranging from 0 (not at all true) to 4 (true nearly all the time), while the total score ranges from 0 to 40. Higher total scores reflect greater resilience. Gabrovec et al. [34] reported adequate reliability (α = 0.89) and construct validity of the Slovenian CD-RISC (CFI = 0.984, SRMR = 0.027, RMSEA = 0.046).

#### 2.3.3. Depression

The presence of depression symptoms during the last two weeks was assessed with The Patient Health Questionnaire (PHQ-9) [41]. This is a widely used screening tool for depression and consists of 9 items describing the DSM-V [42] diagnostic criteria to assess depressive symptomatology on a 4-point Likert scale ranging from 0 (not at all) to 3 (nearly every day), with a total score ranging from 0 to 27. The higher scores indicate more severe depressive symptoms as follows: minimal symptoms (1–4), mild (5–9), moderate (10–14), moderately severe (15–19), and severe (20–27). A score of 10 is the cut-off point used to classify participants exhibiting depressive symptoms. Gabrovec et al. [34] reported adequate reliability (α = 0.91) and construct validity (CFI = 0.991, SRMR = 0.019, RMSEA = 0.047) of the Slovenian PHQ-9.

#### 2.3.4. Anxiety

The Generalized Anxiety Disorder questionnaire (GAD-7) [43] was used to assess the severity of anxiety symptoms. It consists of 7 items which correspond to DSM-IV [44] criteria for generalized anxiety disorder. Participants report how often in the past two weeks they experienced symptoms of anxiety on a 4-point Likert scale from 0 (not at all) to 3 (almost every day). The sum of item scores ranges from 0 to 21; a score of 10 or above indicates the presence of generalized anxiety disorder symptoms. Furthermore, scores of 5, 10 and 15 correspond to mild, moderate and severe anxiety symptoms [43]. Gabrovec et al. [34] reported adequate reliability (α = 0.94) and construct validity of the Slovenian GAD-7 (CFI = 0.996, SRMR = 0.009, RMSEA = 0.049).

#### 2.3.5. Stress

The perceived stress scale (PSS-4) is the shortest version of the three forms of the PSS, developed by Cohen and Williamson [45]. The 4-item scale aims to measure respondents’ coping with stress. Each item is measured on a five-point Likert Scale from 1 (never) to 5 (very often), with two items being reverse coded. Items refer to respondents’ perception of stress in the last month (e.g., “In the last month, how often have you felt that you were unable to control important things in life?”). Although Ingram et al. [46] reported a poor fit of the unidimensional model, the Slovenian PSS-4 data fit the one-factor model well (e.g., CFI = 0.997, SRMR = 0.011, RMSEA = 0.062) as shown in a study by Gabrovec et al. [34]. Internal consistency (Cronbach’s α) of the Slovenian PSS-4 was reported to be 0.80 in the same empirical study.

#### 2.3.6. Other Variables

Other variables included in our study were participants’ gender, and daily social media use during the working days and weekends.

### 2.4. The Model

**Figure 1:**
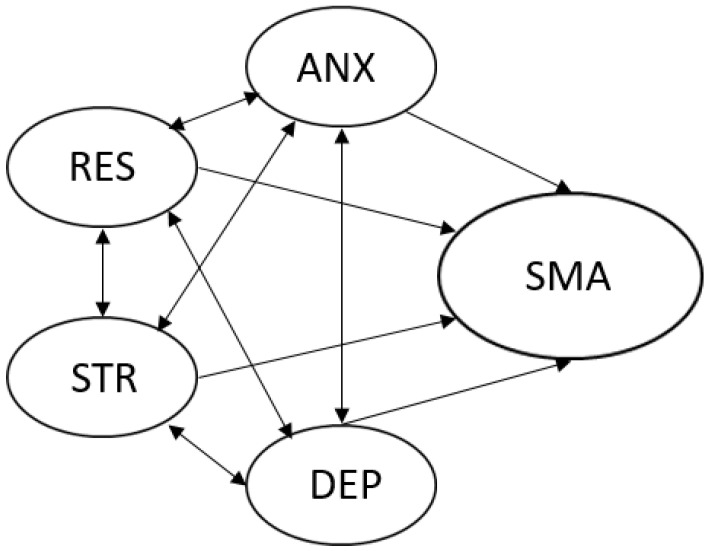
The proposed model of social media addiction (SMA) as a consequence of the interrelationship between resilience (RES), depression symptoms (DEP), anxiety symptoms (AND) and stress symptoms (STR).

The proposed model of social media addiction (SMA) is presented in Figure 1. In the model, resilience (RES), depression symptoms (DEP), anxiety symptoms (ANX) and stress symptoms (STR) are regarded as an interrelated group of variables, related to the state of an individual’s mental health. We acknowledged the possibility of a two-sided effect in each pair of these four variables. Additionally, each of these variables was presumed to affect the likelihood of social media addiction symptoms. This model allowed us to examine multiple indirect effects on social media addiction, where the primary focus was on the indirect effect of resilience on social media addiction through depression symptoms, anxiety symptoms and stress symptoms.

### 2.5. Ethical Considerations

Participants were informed about the purposes of the study and their right to withdraw from the study at any time. They were assured that the data they provided would remain anonymous and be used only for the purposes of scientific research. A written informed consent was obtained from all participants in the present study. Ethical approval to conduct the study was obtained from the National Medical Ethics Committee of the Republic of Slovenia (NMEC), Ministry of Health (No. 0120-48/2021/3).

## 3. Results

### 3.1. Descriptive Statistics of the BSMAS

Means and standard deviations of the BSMAS items and time spent on social media are provided in Table 2. Based on item means in the sample, salience (Item 1) is the most commonly experienced addiction component among social media users, while withdrawal (Item 5) is the least commonly experienced. Notably, the average time spent on social media is close to three hours, regardless of day of the week.

**Figure 2:**
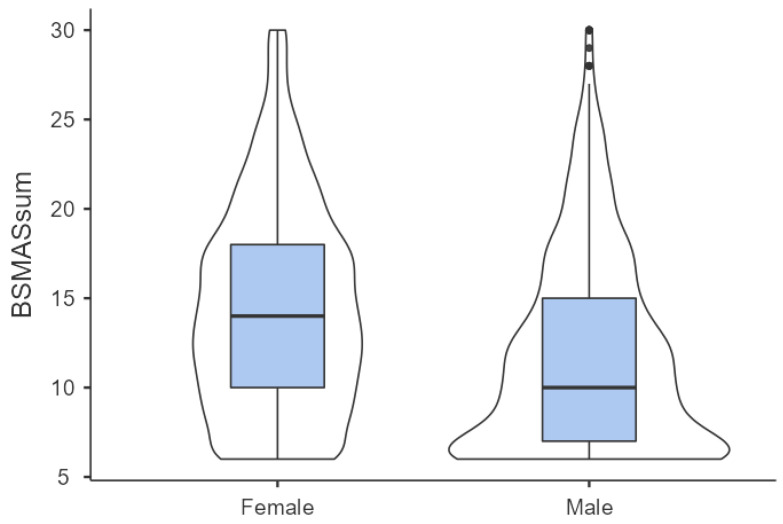
Distribution of total BSMAS scores for both genders.

Differences in BSMAS scores and daily social media use between genders are also shown in Table 2. Females obtained significantly higher total BSMAS scores, as shown by the Mann–Whitney U test (*U* = 1.65 × 10^6^; *p* < 0.001; Cohen’s *d* = 0.46). Females also reported higher daily social media usage both on weekdays (*U* = 1.56 × 10^6^; *p* < 0.001; Cohen’s *d* = 0.51) and on weekends (*U* = 1.62 × 10^6^; *p* < 0.001; Cohen’s *d* = 0.47). In Figure 2, which shows the BSMAS score distribution for both genders, it is worth noting the relatively low proportion of women with the lowest possible score (7.6%), indicating the complete absence of addiction symptoms, compared to men (21.6%).

### 3.2. Prevalence

Based on the proposed cut-off score for social media addiction by Luo et al. [21], i.e., total score of 24 out of 30, the prevalence rate in our sample is 4.5%. The prevalence rate was 2.6% in the male subgroup and 5.3% in the female subgroup, indicating a higher addiction risk for the females. However, the value of Cohen’s *h* = 0.14 is below the commonly reported threshold of 0.2 for small effect.

### 3.3. Reliability

The Slovenian BSMAS demonstrates acceptable reliability (Cronbach’s α = 0.87). Item-level analysis (Table 3) shows that the six BSMAS items are highly and consistently correlated. Potential deletion of any item fails to improve the internal consistency (α) of the scale.

### 3.4. Factorial Validity

To evaluate the unidimensionality and the underlying factor structure of the BSMAS data, PCA was performed. Beforehand, the suitability of the data matrix was explored by Barlett’s test of sphericity (χ² = 13,122; *df* = 15; *p* < 0.001) and the KMO measure of sampling adequacy (KMO = 0.88). The eigenvalue of the first component retained was 3.67, explaining 61.2% of total variance, according to the criterion of parallel analysis. Component loadings for each BSMAS item were between 0.73 (Item 1) and 0.82 (Item 2).

Additionally, the CFA with the DWLS estimation method was performed on a model with one latent factor (Model 1). The model fit was acceptable according to the recommendations by Hooper et al. [47] (Table 4), with slightly elevated values of RMSEA (0.094). However, significant model-fit improvement was found when constraining error terms between Item 1 and Item 2 and between Item 2 and Item 3 (Model 2). Standardized factor loadings for all items in Model 2 were statistically significant (*p* < 0.001) and ranged between 0.66 (Item 1) and 0.85 (Item 4).

### 3.5. Criterion Validity

Correlations between BSMAS scores and key criterion variables are presented in Table 5. Higher BSMAS scores are associated with higher daily social media usage (both on weekdays and weekends) and a higher probability of more symptoms of depression, anxiety and stress. Significant correlations between social media addiction symptoms and the criterion variables are observed at the item level as well. Notably, item 3 (mood modification) shows the strongest correlations with the listed aspects of mental health.

### 3.6. Model Testing

In the first step of evaluating the structural model proposed in Figure 1, which included five latent variables (SMA, RES, ANX, DEP and STR), the measurement model for each latent variable was evaluated. Table 6 contains reliability and fit indices of the five measurement models given by CFA. The presented values refer to fit indices without error-term constraints.

After testing the proposed structural model, we respecified it by constraining error terms in certain item pairs, based on the calculated modification indices. The constraints were made for the following pairs of items: RES1 and RES2, BSMAS1 and BSMAS2, STR2 and STR3, DEP3 and DEP4. Table 7 contains model-fit information for the proposed structural model and its improved version. We find that both models fit the data sufficiently according to recommendations by Hooper et al. [47].

**Figure 3:**
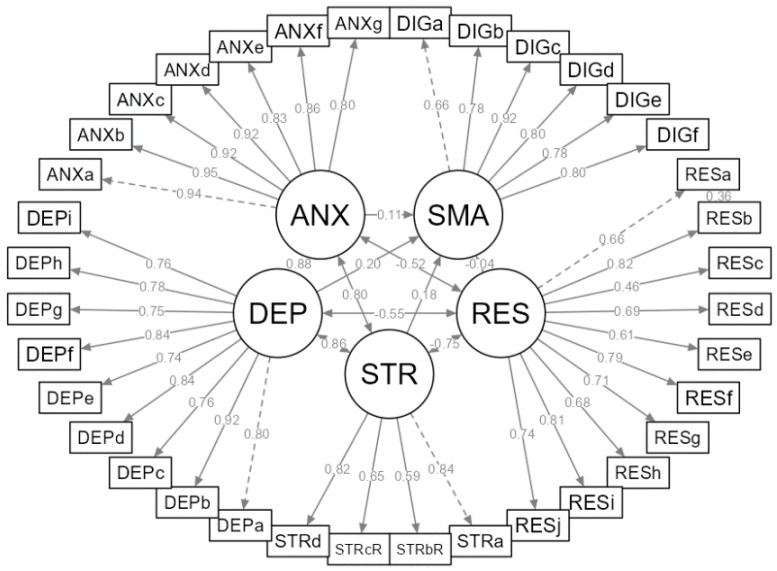
Parameter estimates for the respecified model of social media addiction.

Figure 3 shows the parameter estimates of the respecified model. Examining the standardized path coefficient referring to the direct effects between latent variables, we find that DEP and STR show a significant direct effect on SMA (*β_DEP_* = 0.230, *p* < 0.001, *β_STR_* = 0.171, *p* < 0.001), while RES and ANX do not (*β_RES_* = −0.042, *p* = 0.194, *β_ANX_* = 0.094, *p* = 0.023).

Lastly, we observed indirect effects of RES on SMA through DEP, ANX and STR, shown in Table 8. We find the largest indirect effects of RES on SMA through DEP (*β* = −0.129) and through STR (*β* = −0.128), whereas the indirect effect through ANX is comparatively smaller (*β* = −0.048). Expressed in percentages, indirect effects through DEP, STR and ANX are responsible for 87.8% (37.1% + 36.9% + 13.8%) of the total effect of RES on SMA (*β* = −0.347). The direct effect of RES on SMA therefore only accounts for the remaining 12.2% of the total effect.

## 4. Discussion

The evaluation of the psychometric properties of the Slovenian version of BSMAS, one of the primary aims of the present paper, showed promising results. The instrument displayed high reliability (α = 0.87) with each BSMAS item exhibiting relatively high positive correlations with all other items (0.39 > ρ > 0.64). This indicated that no two items measure completely distinct constructs and that no two items measure the same aspect of the construct. The analysis of factorial validity through PCA revealed the presence of a strong first component, explaining more than 61% of the total item variance. The CFA confirmed that the one-factor measurement model fits the data well, particularly after constraining error terms between Items 1 and 2 and between Items 2 and 3.

Looking at the results of the criterion validity analysis, BSMAS scores are unsurprisingly correlated to daily social media use on weekends and weekdays (ρ = 0.50 for both). Nevertheless, these coefficient values imply that more than half of BSMAS variance remains unexplained after accounting for time spent on social media. Therefore, we can conclude that the Slovenian BSMAS measures much more than just excessive use. As reported in previous BSMAS validation studies [12,15,19], symptoms of depression, anxiety and stress are significantly associated with BSMAS scores. Furthermore, in line with our expectations, resilience negatively predicts BSMAS scores and may play a protective role in the process of developing social media addiction.

These initial findings, which suggest an important role of resilience and mental health in the context of developing social media addiction, were then elaborated on. The proposed structural model, where depression symptoms, anxiety symptoms, stress symptoms and resilience directly and indirectly affect the likelihood of social media addiction symptoms, showed sufficient fit to the data. A significant direct effect on social media addiction was found only for depression and stress symptoms, whereas the direct effects of anxiety and resilience were below the threshold of significance. We speculate that an important causal mechanism behind the two significant direct effects is using social media to (temporarily) alleviate depressive feelings and perceived mental distress. Indeed, among all BSMAS items, the tendency to use social media to forget about personal problems (Item 2—mood modification) is most highly correlated with symptoms of depression and stress in our study.

In the case of resilience, its protective effect on social media addiction was found in a study by Bilgin and Taş [48], while Robertson et al. [49] reported that resilience was a protective factor against internet addiction and gaming addiction, but not for Facebook addiction. In our study, most of its negative effect on social media addiction is indirect (87.2%). In large part, this indirect effect is through a reduction in depressive symptoms and stress symptoms, which in turn reduces the chances of developing social media addiction symptoms. This confirms our hypothesis that resilience, as one of the predictors of mental health during the COVID-19 pandemic, plays a protective role in social media addiction too. Importantly, Wisnievski et al. [50] have also shown that resilience is helpful once internet addiction is already developed; it functions as a protective factor against harms (specifically, negative affect) associated with internet addiction. Taken together, these findings suggest that resilience represents a set of personal qualities which protect the individual both before and after internet addiction. Thus we recognize great potential in further research aiming to prove this hypothesis for social media addiction specifically.

The analysis also encompassed the assessment of social media addiction prevalence. In our sample, which included only tertiary students, the social media addiction prevalence rate was 4.6%. If this is a close approximation of the students presenting with clinically significant symptoms of addiction, it is certainly an alarming figure (albeit not unlikely considering the average daily social media use in the sample is nearly three hours). We acknowledge these figures may be elevated due to self-isolation practices associated with the COVID-19 pandemic. Nevertheless, our estimate is in line with the 5% prevalence figure (based on the most strict cut-off) found by Cheng et al. [8]. Item-level analysis revealed that the most prevalent aspect of addictive use is salience (i.e., preoccupation with social media), whereas the least prevalent aspect is the presence of withdrawal symptoms. The tendency for social media to dominate a person’s thinking and behaviour is therefore the most strongly felt aspect of its problematic use. On the other hand, when a user manages to resist social media for a while, it seems that strong withdrawal symptoms are not particularly common. This is a valuable insight for designing new interventions and strategies aimed at preventing or reducing social media addiction.

Comparisons between genders consistently showed that females were at more risk for social media addiction, which was also found in other studies [17,43]. The prevalence rate in the female subgroup was 5.3% (versus 2.6% in the male subgroup); their BSMAS score was significantly higher on average, and their daily social media use exceeded the daily use in the male subgroup. Interestingly, there were proportionally far fewer females who reported a total absence of social media addiction symptoms compared to males, indicating that women find it harder to maintain “healthy” use of social media. Moreover, women seem to have a greater tendency toward social media addiction, which seems to be in contrast to substance-use addiction or other behavioural addictions [51,52,53].

### Limitations of the Study

A key limitation of the study are the deviations from sample representativeness, such as the disproportionate numbers of female and male participants. Since data collection occurred during the period of the COVID-19 pandemic, when opportunities for offline social contact were limited, generalizing findings (such as daily social media use) to the present time should be done with caution. This calls for the repetition of the research on general population, and in “normal” times. Lastly, the scales used were not simultaneously calibrated with clinical observations.

## 5. Conclusions

In conclusion, the Slovenian BSMAS showed good psychometric properties and may be used in further studies of social media addiction without modifications. The protective role of resilience and the recognition of its indirect effects on social media addiction through depression symptoms and stress are valuable insights, which may be used in preventive efforts against this increasingly prevalent and alarming phenomenon. The reasons behind the apparent gender differences in social media addiction risk should be further explored and may serve as the basis for the development of adapted interventions for each gender.

## Figures and Tables

**Table 1:** Studies evaluating psychometric properties of Bergen Social Media Addiction Scale.

First Author	Year of Publication	Country	*N*	Mean Age (SD) in Years	Reliability (Cronbach’s α)	Factor Analyses
Banyai	2017	Hungary	5961	16.6 (1.0)	0.85	CFA—acceptable model fit
Lin	2017	Iran	2676	15.5 (1.2)	0.86	CFA—acceptable model fit
Monacis	2017	Italy	769	21.6 (3.9)	0.88	CFA—acceptable model fit
Leung	2020	China, Taiwan	306, 336	24.1 (5.1), 20.5 (1.2)	0.85	CFA—acceptable model fit
Dadiotis	2021	Greece	325	21.6 (5.3)	0.75	CFA—acceptable model fit
Brailovskaia	2022	China, France, Germany, Poland, Russia, Spain, Sweden, U.K., U.S.	9418	18+ *	0.81–0.90	EFA—unidimensional structure
Shin	2022	South Korea	401	21.9 (1.8)	0.86	CFA—acceptable model fit
Stănculescu	2022	Romania	705	30.2 (9.1)	0.84	CFA—acceptable model fit

CFA = confirmatory factor analysis * Mean age and standard deviation not reported.

**Table 2:** Means and standard deviations for each BSMAS item, total score and social media use with gender differences and effect sizes.

	How Often during the Past Year Have You…	Addiction Component	M(SD) All	M(SD) Male (*n* = 1306)	M(SD) Female (*n* = 3524)	ES
Item 1	…spent a lot of time thinking about social media or planned use of social media?	salience	2.72 (1.26)	2.34 (1.23)	2.86 (1.24)	0.37
Item 2	…felt an urge to use social media more and more?	tolerance	2.47 (1.28)	2.11 (1.21)	2.61 (1.27)	0.36
Item 3	…used social media in order to forget about personal problems?	mood modification	2.5 (1.34)	2.04 (1.21)	2.67 (1.35)	0.41
Item 4	…tried to cut down on the use of social media without success?	relapse	2.15 (1.20)	1.87 (1.11)	2.25 (1.22)	0.28
Item 5	…become restless or troubled if you have been prohibited from using social media?	withdrawal	1.61 (0.98)	1.46 (0.87)	1.66 (1.01)	0.17
Item 6	…used social media so much that it has had a negative impact on your job/studies?	conflict	2.24 (1.28)	2.36 (1.30)	2.36 (1.30)	0.31
Total score (BSMAS)			13.70 (5.75)	11.74 (5.48)	14.42 (5.68)	0.46
Daily social media use (minutes)—Monday-Friday			162.5 (128.5)	127.3 (106.0)	175.61 (133.5)	0.51
Daily social media use (minutes)—Saturday-Sunday			177.6 (136.3)	144.2 (124.7)	190.0 (138.3)	0.47

Note. ES = effect size of differences between genders calculated from *U* and reported as Cohen’s *d*.

**Table 3:** Alpha if item deleted and correlations with other items for each BSMAS item.

			Correlation Matrix (Spearman’s Rho)				
	α If Item Deleted	Item-Total r	Item 1	Item 2	Item 3	Item 4	Item 5
Item 1	0.86	0.62					
Item 2	0.84	0.72	0.64				
Item 3	0.84	0.70	0.50	0.61			
Item 4	0.84	0.71	0.46	0.56	0.59		
Item 5	0.86	0.63	0.39	0.48	0.49	0.57	
Item 6	0.85	0.67	0.44	0.51	0.54	0.59	0.52

**Table 4:** Goodness-of-fit indices and evaluations for the two models.

	χ^2^	df	*p*-Value	CFI	TLI	RMSEA	SRMR	Overall Evaluation
Recommendations by Hooper et al.	/	/	>0.05	>0.90	>0.90	<0.10	<0.10	Acceptable fit
	/	/	>0.05	>0.95	>0.95	<0.08	<0.08	Good fit
Model 1	394	9	<0.001	0.994	0.989	0.094	0.038	Acceptable fit
Model 2	30.8	7	<0.001	1.000	0.999	0.026	0.013	Good fit

CFI = comparative fit index; TLI = Tucker–Lewis index; RMSEA = root mean square error of approximation; SRMR = standardized root mean square residual.

**Table 5:** Correlation coefficients (Spearman’s ρ) between BSMAS score and key criterion variables.

	Addiction Component	SM Usage (Weekday)	SM Usage (Weekend)	DEP	ANX	STR	RES
Item 1	salience	0.41 *	0.41	0.21	0.23	0.18	−0.14
Item 2	tolerance	0.41	0.40	0.28	0.29	0.25	−0.19
Item 3	mood modification	0.39	0.41	0.44	0.43	0.41	−0.32
Item 4	relapse	0.37	0.38	0.30	0.29	0.27	−0.25
Item 5	withdrawal	0.32	0.34	0.27	0.25	0.22	−0.22
Item 6	conflict	0.40	0.39	0.35	0.30	0.33	−0.26
BSMAS score		0.50	0.50	0.40	0.39	0.36	−0.29

* all correlations are significant at *p* < 0.001.

**Table 6:** Descriptive statistics, reliability and model-fit indices of five latent variables.

Latent Variable	Mean Total Score	SD	Cronbach’s Alpha	CFI	TLI	RMSEA	SRMR
SMA	13.7	5.75	0.87	0.994	0.989	0.094	0.043
ANX	10.4	6.55	0.94	1.000	0.999	0.049	0.019
DEP	11.3	7.25	0.91	0.996	0.994	0.068	0.040
STR	8.0	3.31	0.80	0.976	0.928	0.286	0.070
RES	23.6	7.37	0.89	0.992	0.990	0.072	0.041

SMA = social media addiction, RES = resilience, DEP = depression symptoms, ANX = anxiety symptoms, STR = stress symptoms.

**Table 7:** Fit indices of the hypothesized and respecified model.

	Hypothesized Model	Respecified Model
χ^2^	8501	6233
df	584	580
*p*	<0.001	<0.001
CFI	0.994	0.995
TLI	0.993	0.995
RMSEA	0.054	0.046
SRMR	0.043	0.039

**Table 8:** Total, direct and indirect effects on SMA and their size.

Effect Type	Effect	β
Total effect	RES on SMA	−0.347
Direct effect	DEP on SMA	0.230
Direct effect	STR on SMA	0.171
Direct effect	RES on SMA	−0.042
Direct effect	ANX on SMA	0.094
Indirect effect	RES on SMA through DEP	−0.129
Indirect effect	RES on SMA through STR	−0.128
Indirect effect	RES on SMA through ANX	−0.048

## Data Availability

Supporting results can be found at: https://www.nijz.si/sl/ukrepi-na-podrocju-obvladovanja-siritve-covid-19-s-poudarkom-na-ranljivih-skupinah-prebivalstva (accessed on 11 October 2021).
